# Hydrogel coils versus bare platinum coils for the endovascular treatment of intracranial aneurysms: a meta-analysis of randomized controlled trials

**DOI:** 10.1186/s12883-018-1171-8

**Published:** 2018-10-05

**Authors:** Tao Xue, Zhouqing Chen, Weiwei Lin, Jiayi Xu, Xuming Shen, Zhong Wang

**Affiliations:** 1 Department of Neurosurgery & Brain and Nerve Research Laboratory, The First Affiliated Hospital of Soochow University, 188 Shizi Street, Suzhou, 215006 China; 2 University of Pittsburgh School of Pharmacy, Pittsburgh, PA 15219 USA; 3 Department of Ophthalmology, The First Affiliated Hospital of Soochow University, Suzhou, 215006 Jiangsu Province China; 4 Department of Neurosurgery, Taicang Affiliated Hospital of Soochow University, Suzhou, 215400 Jiangsu Province China

**Keywords:** Hydrogel coils, Bare platinum coils, Endovascular treatment, Intracranial aneurysms, Meta-analysis

## Abstract

**Background:**

Recent studies have shown conflicting results regarding the effect of hydrogel coils for treating intracranial aneurysm compared to bare platinum coils. We implemented a meta-analysis to assess the value of hydrogel coils in intracranial aneurysm treatment.

**Methods:**

The MEDLINE, EMBASE, and Cochrane Library databases were searched for randomized controlled trials (RCTs) which had evaluated hydrogel coils versus bare platinum coils for intracranial aneurysms.

**Results:**

We pooled 1526 patients from 4 RCTs with the mean follow-up time of more than 16 months. Hydrogel coils had reductions on mid-term recurrence (RR 0.78, 95% CI 0.65 to 0.94, *P* = 0.008) and residual aneurysm (RR 0.71, 95% CI 0.57 to 0.88, *P* = 0.002), but didn’t show any significant differences in other favorable outcomes such as functional recovery, mortality and so on. In the subgroup analysis, we found that second-generation hydrogel coils might exhibit potential impacts on increasing mid-term complete occlusion (RR 1.26, 95% CI 1.07 to 1.48, *P* = 0.005) and decreasing residual aneurysm neck. (RR 0.54, 95% CI 0.34 to 0.86, *P* = 0.010).

**Conclusions:**

Hydrogel coils showed no significant efficacy on functional recovery but exhibited a lower rate of recurrences and residual aneurysms in patients with intracranial aneurysms.

**Electronic supplementary material:**

The online version of this article (10.1186/s12883-018-1171-8) contains supplementary material, which is available to authorized users.

## Background

Worldwide, intracranial aneurysm, as a life-threatening disease with a high morbidity and mortality rate, brings great economic burdens to both the society and the patient [1]. Intracranial aneurysm is the third most common type of stroke, after ischemic stroke and hypertensive cerebral hemorrhage, which plays the most important role in subarachnoid hemorrhage [2].

Before 1990, neurosurgical operations were the only option for patients with intracranial aneurysm [3]. At 1992, Guglielmi detachable platinum coils(GDC) were introduced as an endovascular treatment which provided patients with an additional treatment option [4–6]. Endovascular coil emblization eventually became the preferred modality for many patients, due to better clinical outcomes than neurosurgical clipping in some patients with intracranial aneurysm demonstrated in ISAT(International Subarachnoid Aneurysm Trial) [7]. Although endovascular treatment is a less invasive procedure than neurosurgical aneurysm clipping, patients with endovascular treatment have a higher rate of aneurysm remnant, recurrence and retreatment than patients treated by clipping, in spite of the overall incidence is low [8–11]. Hence, there still exists a need for improvement in methods of embolization to reduce incomplete occlusion, recurrence, retreatment and post-treatment adverse events [4, 12, 13].

Hydrogel embolic system(HES) is made up of platinum coils covered with cross-linked material, which can absorb much water without dissolving, and presents both liquid-like and solid-like softening behaviors [4, 14]. Therefore, it may provide better efficacy and safety in endovascular treatment. The first-generation hydrogel-coated coils (hydroCoil; MicroVention, Inc., Tustin,CA) [15] were assessed in some clinical trials to compare the clinical outcomes of hydrogel coils and bare platinum coils. These clinical trials include HELPS (Hydrocoil Endovascular aneurysm occLusion and Packing Study) [16] and Poncyljusz’s RCT [17]. HELPS demonstrated that hydrogel coils have a reduction of adverse events and recurrence among intracranial aneurysm patients [18]. In contrast, Poncyljusz’s RCT concluded that hydrogel coils were equally as effective as bare platinum coils [17]. Later, the second-generation hydrogel (Hydrosoft, HydroFrame[3D], MicroVention, Inc) was developed, which was softer, contained less hydrogel, and expanded slower than the first-generation one [19]. In 2018, GREAT(German-French Randomized Endovascular Aneurysm Trial) was established, and some researchers found that compared to bare platinum coils, hydrogel coils decrease the rate of unfavorable outcome events, recurrence, retreatment, morbidity mortality, and need for retreatment in small- and medium-sized intracranial aneurysms [19, 20]. Containing both types of hydrogel coils, PRET 2017(Patients prone to Recurrence after Endovascular Treatment) illustrated that there were no significant differences between hydrogel coils group and bare platinum coils group [21].

Based on the above-mentioned results from previous clinical studies and trials, the efficacy and safety of hydrogel coils treatment for intracranial aneurysm are unclear. Several issues still need to be resolved, including whether or not the use of hydrogel coils reduces incomplete occlusion, complications, adverse events, recurrence, retreatment, morbidity, mortality, etc. We present a meta-analysis of pooled data from previous clinical trials to investigate the value of hydrogel coils treatment for intracranial aneurysm and to explore the potential factors that might influence the efficacy and safety of hydrogel coils.

## Methods

### Study protocol

A research protocol was drafted following the Cochrane Collaboration format at the beginning of the project [22].

### Eligibility criteria

The inclusion criteria were as follows: (a) Type of study: RCT; (b) Language restriction: only available in English; (c) Participating patients: patients with intracranial aneurysms; (d) Intervention: Hydrogel coils or bare platinum coils; (e) Outcomes: complete occlusion, residual neck and residual aneurysm on DSA, excellent outcome (mRS score = 0) and favorable outcome (mRS score = 0–2) based on mRS score, periprocedural complications, major recurrence, retreatment and adverse events. The exclusion criteria were as follows: (a) Types of study: retrospective studies, cohort studies, case reviews and case reports; (b) Control: positive control.

### Information sources and search strategy

Three main databases: EMBASE, MEDLINE and Cochrane Library were systematically searched by three authors (TX, ZC and JX). The search strategy was a combination of the variables “coil” AND “intracranial aneurysm” for MEDLINE. Only studies that match the titles and abstracts were searched. The search strategy of Cochrane Library and EMBASE were similar to that search strategy of MEDLINE. In addition, two investigators (TX and ZC) ensured all relevant studies included in the study. They independently manually screened the list of references from the RCTs and systematic reviews.

### Study selection and data collection

Two reviewers (TX and ZC) independently assessed all study records from systematic search in systematic reviews and reference lists of RCTs and electronic database on the previously mentioned the eligbility criteria. After the rigorous selection and evaluation of the literature by the two reviewers, the data were extracted from the included RCTs as follows: basic information for included RCTs, inclusion, exclusion criteria, study design and outcome assessments (Table 1).

**Table 1:** Characteristics of the Included Studies and Outcome Events

Trials	GREAT 2018 (DRKS00003132)	PRET 2017 (NCT00626912)	Poncyljusz’s RCT 2014 (EURR-6928)	HELP 2011 (ISRCTN30531382)
*Regions*	22 centers in 2 countries	25 centers in 6 countries	1 centers in 1 country	24 centers in 7 countries
*Publication*	Stroke	Am J Neuroradiol	European Journal of Radiology	Lancet
*Inclusion Criteria*	Ruptured or unruptured IAs; WFNS grade: 0–3; Age: 18–75 years; IA size: 4-12 mm in diameter; Endovascular occlusion is deemed possible; Neurointerventionist is content to use either HC or BPC.	Ruptured or unruptured IAs; WFNS grade: 0–3; Age: > 18 years; Life expectancy: > 2 years Endovascular occlusion is considered possible by both coils; Neurointerventionist is satisfied with using either HC or BPC but not other type.	Only unruptured IAs; Endovascular occlusion is considered possible by both coils; Neurointerventionist is satisfied with using either HC or BPC but not other type.	Ruptured or unruptured IAs; Previously untreated IAs; Not pregnant; WFNS grade: 0–3; Age: 18–75 years; IA size: 2-25 mm in maximum diameter; Endovascular occlusion is deemed possible; Neurointerventionist is content to use either HC or BPC.
*Exclusion Criteria*	Patients already randomized in this trials; Pre-treated IA by coiling or clipping; More than one IAs need to be treated at the same treatment episode.	Other IAs requiring to be treated at the same treatment episode; Presence of AVM; Absolue contraindication to endovascular treatment.	Ruptured IAs; Intolerance to heparin or resistance to antiplatelet therapy, coagulopathies and abnormal platelet outcome.	Patients already randomized in this trials; More than one IAs need to be treated at the same treatment episode.
*Study Design*	Second-generation hydrogel coil (HydroSoft and/or HydroFrame) vs. Bare platinum coil	First or Second-generation hydrogel coil vs. Bare platinum coil	Hydrogel-coated coil vs. Bare platinum coil	First-generation hydrogel coil vs. Bare platinum coil
*Efficacy outcomes*	Complete occlusion, residual neck and residual aneurysm at periprecedure and 6–18 months; Recurrence, retreatment and mRS responese at 6–18 months.	Complete occlusion, residual neck and residual aneurysm at periprecedure; Recurrence, retreatment and mRS responese at 18 months.	Complete occlusion, residual neck and residual aneurysm at periprecedure and 12 months; Recurrence, retreatment and mRS responese at 12 months.	Complete occlusion, residual neck and residual aneurysm at periprecedure and 18 months; Recurrence, retreatment and mRS responese at 18 months.
*Safety outcomes*	Thromboembolic complications, coil migration, peforation, etc. at periprecedure; AEs, SAEs and death at periprocedure and 6-18 months.	Thromboembolic complications, hydrocephalus, peforation, etc. at periprecedure; AEs, SAEs and death at periprocedure and 18 months.	Thromboembolic complications, hydrocephalus, cerebral edema, etc. at periprecedure; AEs and death at periprocedure and 12 months.	Thromboembolic complications, artery occlusion, peforation, etc. at periprecedure; AEs and death at periprocedure and 18 months.

*GREAT* German-French Randomized Endovascular Aneurysm Trial, *PRET* Patients prone to Recurrence after Endovascular Treatment, *HELP* Hydrocoil Endovascular aneurysm occLusion and Packing Study, *WFNS* World Federation of Neurosurgeons Societies, *IA* Intracranial aneurysm, *AEs* Adverse Events, *SAEs* Severe Adverse Events, *AVM* arteriovenous malformation, *HC* hydrogel coil, *BPC* Bare platinum coil

### Risk of Bias

The risk of bias plot was based on the Review Manager 5.2 software for individual studies. We applied the unified standard of the Cochrane Collaboration to assess the risk of bias of RCTs, which included: selection bias, performance bias, detection bias, attrition bias, reporting bias, and other potential biases.

### Summary measures and synthesis of results

The data was assessed by STATA (Version 12.0) software. The risk ratio (relative risk [RR]; 95% confidence interval [CI]) was analyzed using dichotomous outcomes and calculated using a random effect model. Heterogeneity was estimated by the *I*^2^ statistic. The *I*^2^ statistic as follows: *I*^2^ < 30% means “low heterogeneity”; *I*^2^ = 30 to 50% denotes “moderate heterogeneity”; *I*^2^ > 50% represents “substantial heterogeneity”. Subgroup analyses were implemented to detect different generations hydrogel coils and ruptured rate of aneurysm at baseline. Explore the stability of the consolidated results using sensitivity analysis. A *P* value of less than 0.05 was considered to be significant and two-tailed tests were implemented for all analyses.

## Results

**Fig. 1:**
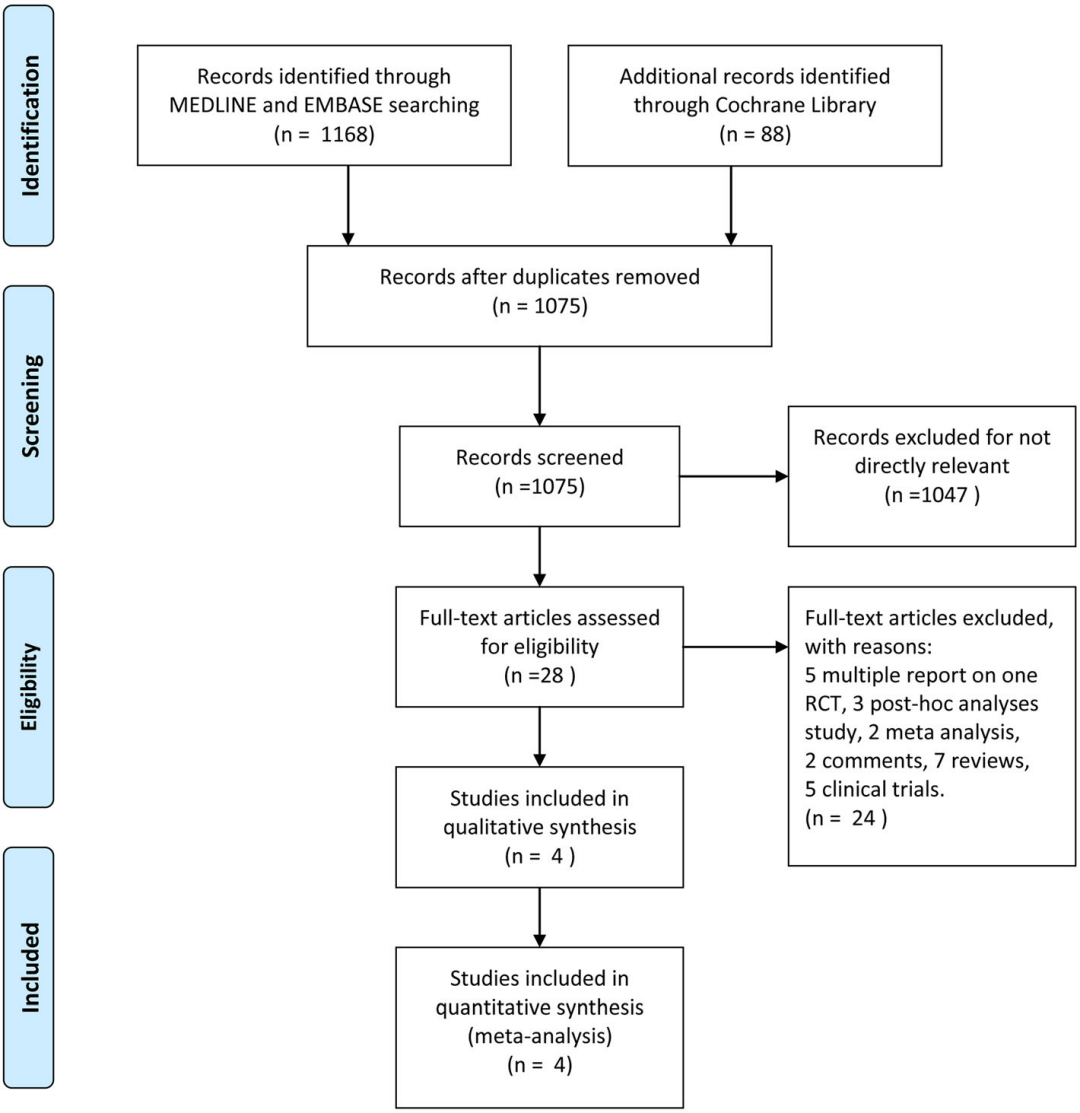
The study search, selection, and inclusion process

A total of 1256 titles and abstracts were identified through MEDLINE, EMBASE, and Cochrane Library (Fig. 1). After removing the duplicates and irrelevant records, 28 full-text articles were assessed for eligibility. Additionally, 24 articles were excluded as a result of the limitation of publication types: 5 multiple reports on one RCT, 3 post-hoc analysis, 2 meta-analysis, 2 comments, 7 reviews and 5 clinical trials. Ultimately, four RCTs containing 1526 patients (hydrogel, *n* = 767; bare, *n* = 759) were included in qualitative synthesis (Fig. 1). The main characteristics of the included studies are listed in Table 1.

### Outcomes analysis

All 4 RCTs [16, 17, 19, 21] enrolling 1526 patients were pooled for the analysis of periprocedural and mid-term outcome respectively from two aspects of efficacy and safety.

#### Periprocedural efficacy and safety outcome

**Fig. 2:**
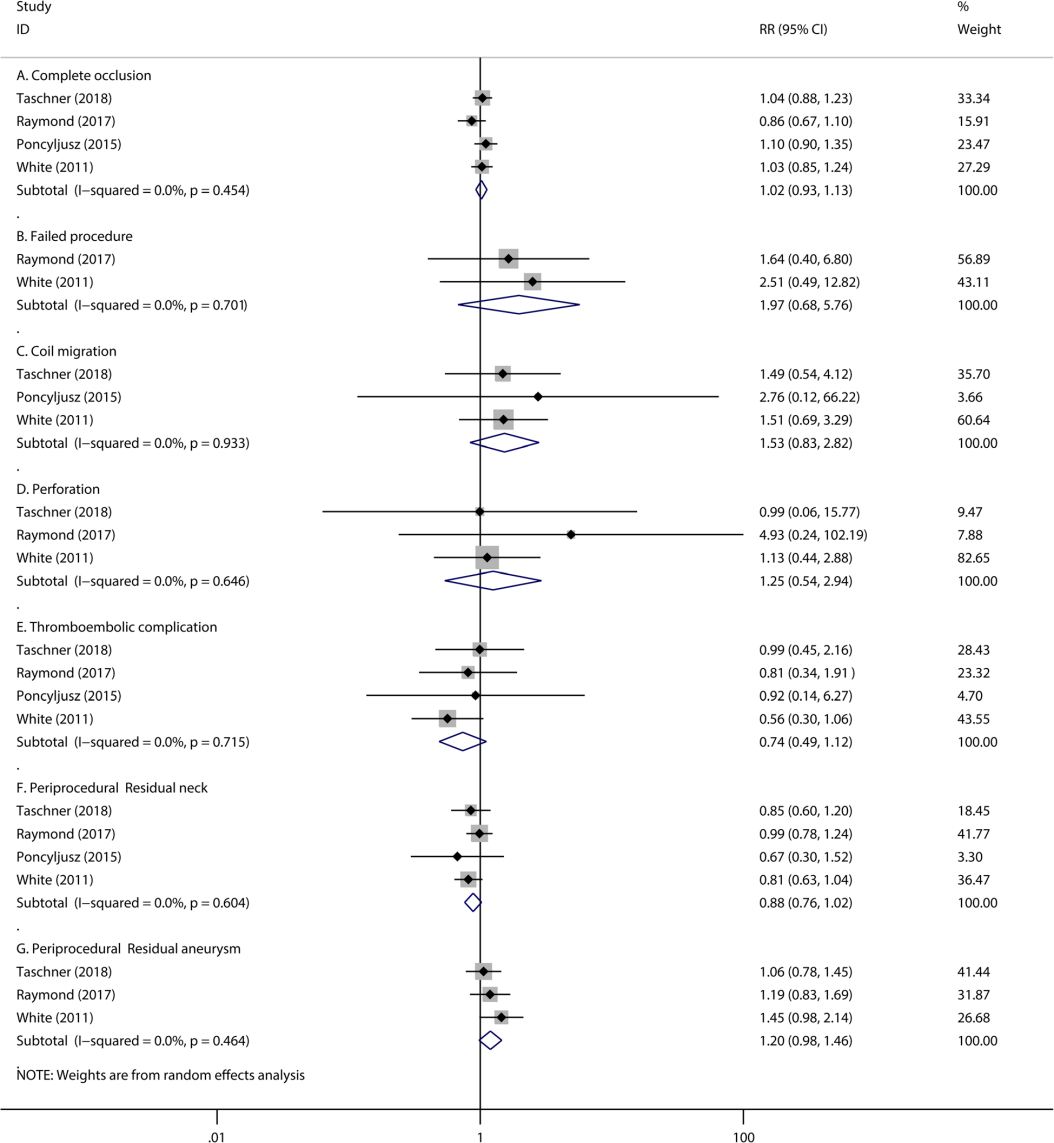
The pooled relative risk of the periprocedural efficacy and safety outcome. The diamond indicates the estimated relative risk (95% confidence interval) for all patients together. A, Complete occlusion. B, Failed procedure. C, Coil migration. D, Perforation. E, Thromboembolic complication. F, Periprocedural residual neck. G, Periprocedural residual aneurysm

**Fig. 3:**
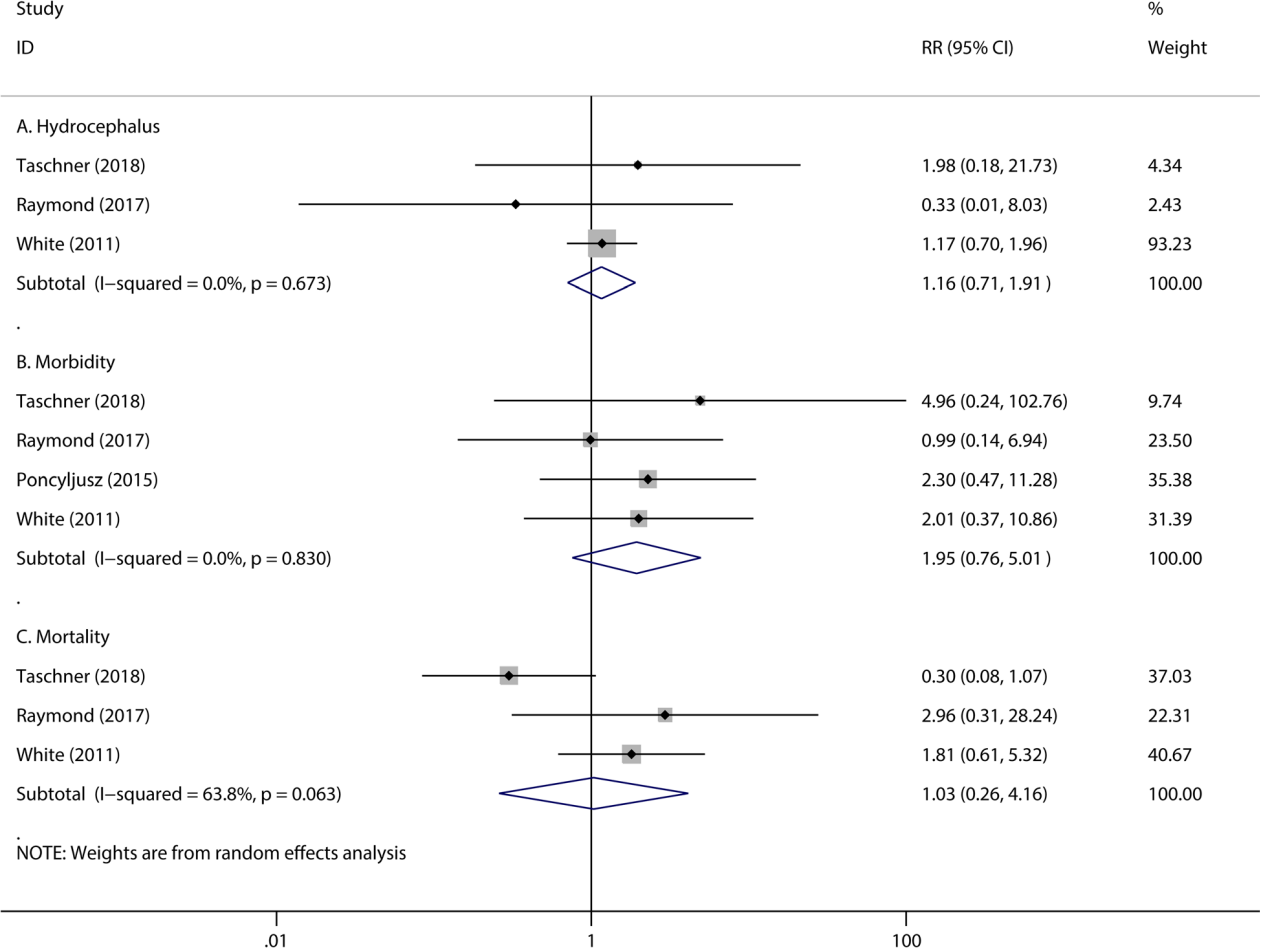
The pooled relative risk of the periprocedural safety outcomes. The diamond indicates the estimated relative risk (95% confidence interval) for all patients together. A, Hydrocephalus. B, Morbidity. C, Mortality

There were no significant differences observed in the numbers of patients with initial complete occlusion after endovascular treatment (RR 1.02, 95% CI 0.93 to 1.13, *P* = 0.679; Fig. 2A) between the hydrogel coil and bare platinum coil groups. Compared with bare platinum coils, hydrogel coils showed no significant differences in preventing periprocedural events, including: failed procedure (RR 1.97, 95% CI 0.68 to 5.76, *P* = 0.213, Fig. 2B), coil migration (RR 1.53, 95% CI 0.83 to 2.82, *P* = 0.168, Fig. 2C), perforation (RR 1.25, 95% CI 0.54 to 2.94, *P* = 0.603, Fig. 2D), hydrocephalus (RR 1.16, 95% CI 0.71 to 1.91, *P* = 0.555, Fig. 3A) and thromboembolic complications (RR 0.74, 95% CI 0.49 to 1.12, *P* = 0.148, Fig. 2E). The numbers of periprocedural residual aneurysm neck (RR 0.88, 95% CI 0.76 to 1.02, *P* = 0.098, Fig. 2F) and residual aneurysm (RR 1.20, 95% CI 0.98 to 1.46, *P* = 0.080, Fig. 2G) were also similar in the two groups; In addition, morbidity (RR 1.95, 95% CI 0.76 to 5.01, *P* = 0.167, Fig. 3B) or mortality (RR 1.03, 95% CI 0.26 to 4.16, *P* = 0.962, Fig. 3C) also showed no significant difference. The heterogeneity of periprocedural mortality is 63.8% with a *P* value of 0.063 (Fig. 3C). To detect the source of this statistical heterogeneity, a sensitivity analysis was performed. The sensitivity analysis showed that all of the consolidated results were stable (Additional file 1: Figure S1).

#### Mid-term efficacy outcome

**Fig. 4:**
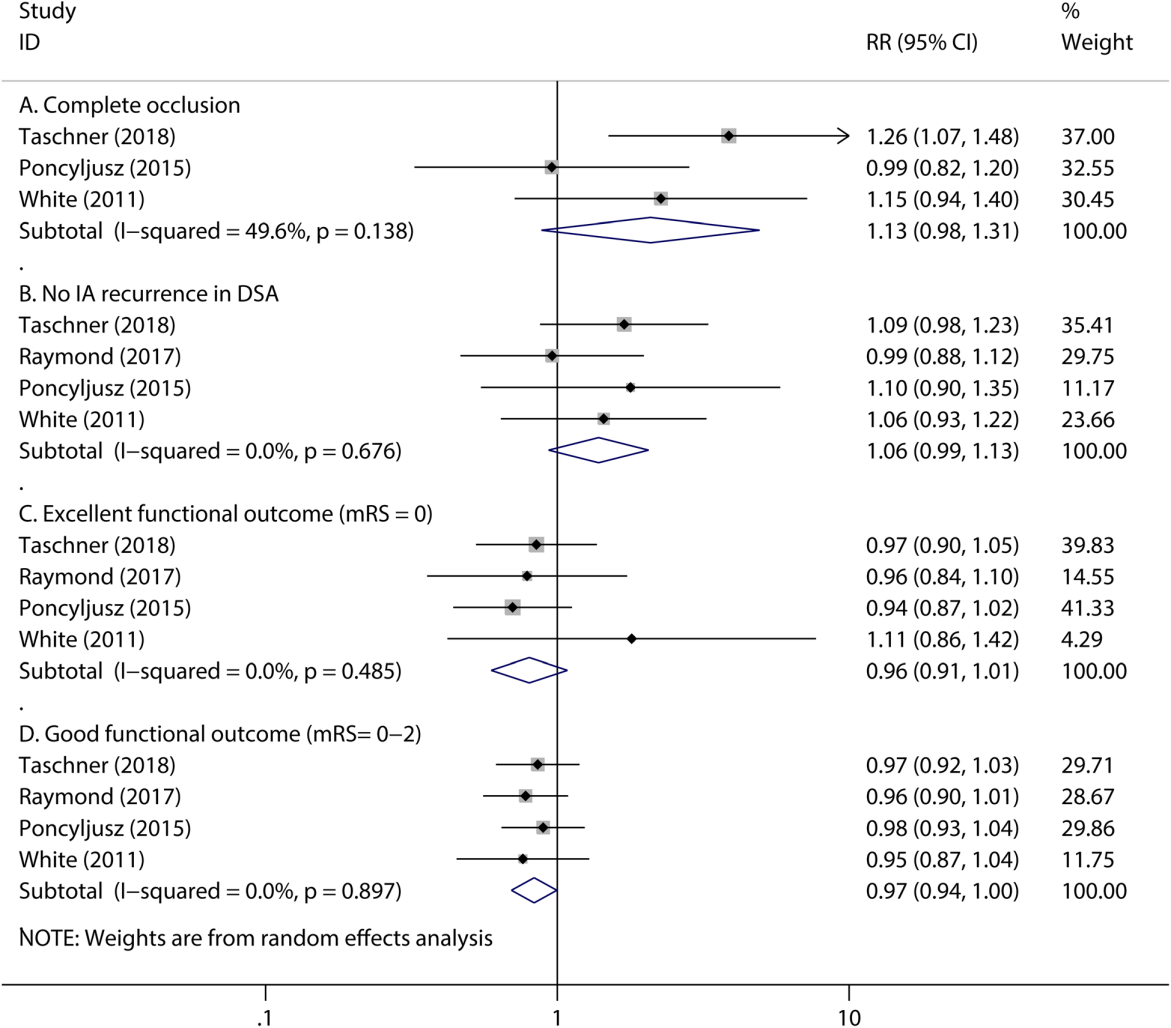
The pooled relative risk of the mid-term efficacy outcomes. The diamond indicates the estimated relative risk (95% confidence interval) for all patients together. A, Complete occlusion. B, No IA recurrence in DSA. C, Excellent functional outcome (mRS = 0). D, Good functional outcome (mRS = 0–2)

Although no significant differences exist in the numbers of mid-term complete occlusion (RR 1.13, 95% CI 0.98 to 1.31, *P* = 0.094; Fig. 4A), no intracranial aneurysms recurrences in DSA (RR 1.06, 95% CI 0.99 to 1.13, *P* = 0.106; Fig. 4B) and excellent functional outcome (mRS score = 0) (RR 0.96, 95% CI 0.91 to 1.01, *P* = 0.150; Fig. 4C) for both groups, bare platinum coils group have an advantage over hydrogel coils group in patients’ good functional outcome(RR 0.97, 95%CI 0.94 to 1.00, *P* = 0.046, Fig. 4D), which is defined as the follow-up mRS score from 0 to 2. The heterogeneity of mid-term complete occlusion is 49.6% with a *P* value of 0.138(Fig. 4A). To detect the source of the statistical heterogeneity, a sensitivity analysis was performed. The sensitivity analysis showed that all of the consolidated results were stable (Additional file 1: Figure S2).

#### Mid-term safety outcome

**Fig. 5:**
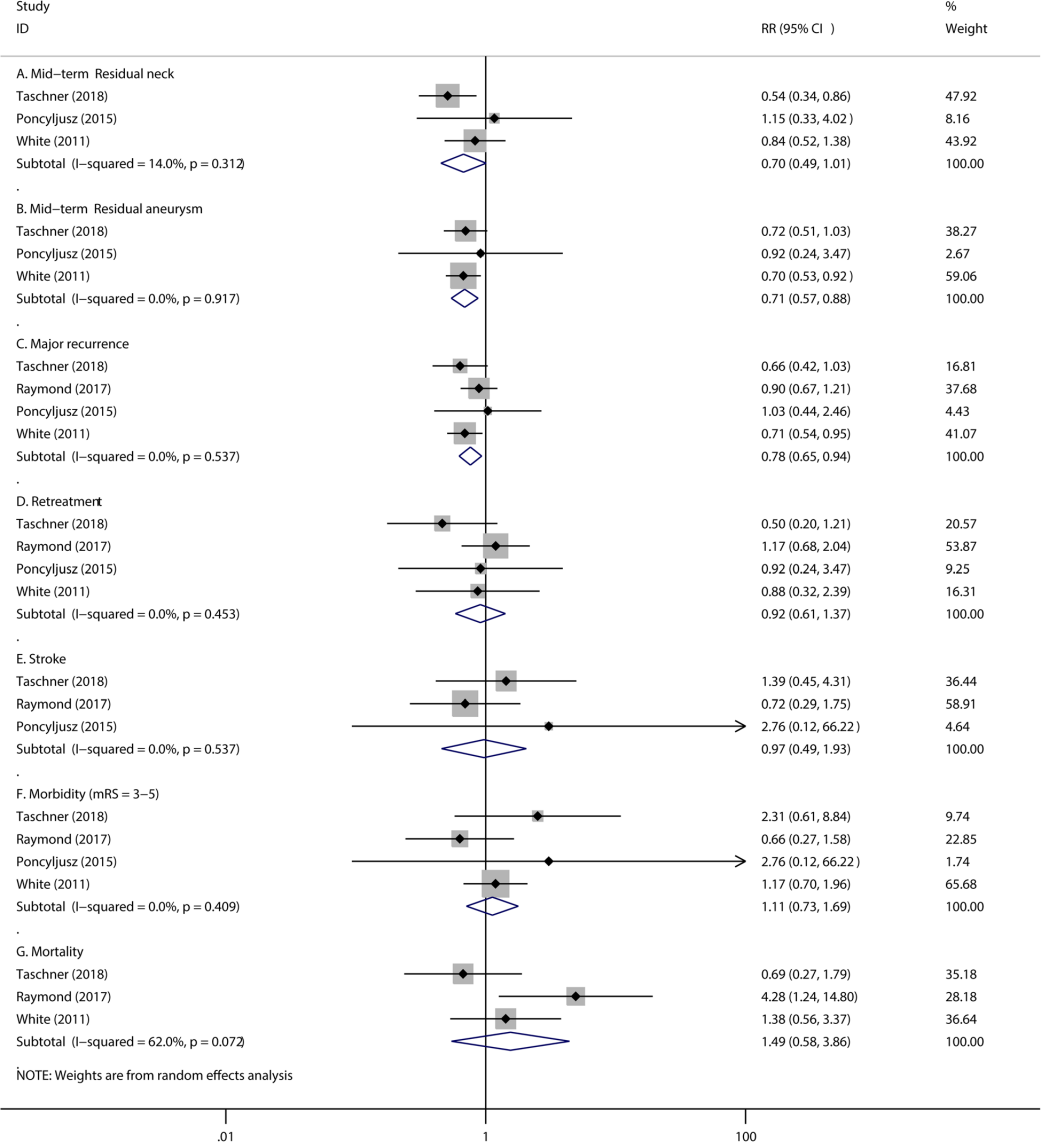
The pooled relative risk of the mid-term safety outcomes. The diamond indicates the estimated relative risk (95% confidence interval) for all patients together. A, Mid-term Residual neck. B, Mid-term Residual aneurysm. C, Major recurrence. D, Retreatment. E, Stroke. F, Morbidity (mRS = 3–5). G, Mortality

Patients in the hydrogel coils group have a lower rate of mid-term residual neck (RR 0.70, 95% CI 0.49 to 1.01, *P* = 0.054; Fig. 5A), residual aneurysm (RR 0.71, 95% CI 0.57 to 0.88, *P* = 0.002; Fig. 5B) and major recurrence (RR 0.78, 95% CI 0.65 to 0.94, *P* = 0.008; Fig. 5C) than that of the bare platinum coils group. However, this was accompanied by no reduction in the need for retreatment (RR 0.92, 95% CI 0.61 to 1.37, *P* = 0.675; Fig. 5D), stroke (RR 0.97, 95% CI 0.49 to 1.93, *P* = 0.934; Fig. 5E), morbidity (RR 1.11, 95% CI 0.73 to 1.69, *P* = 0.615; Fig. 5E) and mortality (RR 1.49, 95% CI 0.58 to 3.86, *P* = 0.411; Fig. 5G) after hydrogel coil treatment compared to bare coil treatment. The heterogeneity of Mid-term mortality is 62.0% with *P* value of 0.072 (Fig. 5G). To detect the source of this statistical heterogeneity, sensitivity analysis was performed. The sensitivity analysis showed that all of the consolidated results were stable (Additional file 1: Figure S3).

### Subgroup analysis

Subgroup analyses were implemented to assess the influence of different generations of hydrogel coils and ruptured rate of aneurysm at baseline. Second-generation hydrogel coils were more effective in mid-term complete occlusion (RR 1.26, 95% CI 1.07 to 1.48, *P* = 0.005; Table. 2) and had a lower rate of residual neck (RR 0.54, 95% CI 0.34 to 0.86, *P* = 0.010; Table. 2). For high ruptured rate subgroup, in which each trial’s proportion of ruptured aneurysms was more than 30%, intracranial aneurysms were more likely to be occluded completely by hydrogel coils than bare platinum coils (RR 1.21, 95% CI 1.07 to 1.38, *P* = 0.002; Table. 2). The sensitivity analysis demonstrated that all the consolidated statistics were stabilized.

**Table 2:** Subgroup Analysis of Efficacy and Safety Outcomes

	Efficacy outcomes							
	Complete occlusion		Recurrence in DSA		Excellent functional outcome		Good functional outcome	
	RR (95% CI)	*P* value	RR (95% CI)	*P* value	RR (95% CI)	*P* value	RR (95% CI)	*P* value
1. Hydrogel coil								
Second generation	1.26 (1.07, 1.48)	0.005	1.09 (0.98, 1.23)	0.119	0.97 (0.90, 1.05)	0.498	0.97 (0.92, 1.03)	0.358
First generation	1.07 (0.90, 1.25)	0.450	1.04 (0.95, 1.13)	0.390	0.97 (0.88, 1.06)	0.482	0.97 (0.93, 1.00)	0.074
2. Ruptured ratio (%)								
*N* > 30	1.21 (1.07, 1.38)	0.002	1.08 (0.99, 1.18)	0.077	1.00 (0.88, 1.12)	0.964	0.97 (0.92, 1.02)	0.187
*N* < 30	0.99 (0.82, 1.20)	0.938	1.02 (0.92, 1.18)	0.684	0.95 (0.88, 1.01)	0.116	0.97 (0.93, 1.01)	0.133
	Safety outcomes							
	Mid-term residual neck		Retreatment		Stroke		Morbidity	
	RR (95% CI)	*P* value	RR (95% CI)	*P* value	RR (95% CI)	*P* value	RR (95% CI)	*P* value
3. Hydrogel coil								
Second generation	0.54 (0.34, 0.86)	0.010	0.50 (0.20, 1.21)	0.122	1.39 (0.45, 4.31)	0.570	2.31 (0.61, 8.84)	0.220
First generation	0.88 (0.56, 1.39)	0.576	1.08 (0.68, 1.69)	0.752	0.79 (0.34, 1.87)	0.594	1.03 (0.66, 1.60)	0.899
4. Ruptured ratio (%)								
*N* > 30	0.67 (0.43, 1.03)	0.070	0.64 (0.33, 1.24)	0.186	1.39 (0.45, 4.31)	0.570	1.28 (0.79, 2.07)	0.317
*N* < 30	1.15 (0.33, 4.02)	0.827	1.13 (0.68, 1.88)	0.629	0.79 (0.34, 1.87)	0.594	0.73 (0.31, 1.69)	0.461

### Risk of Bias in included studies

**Fig. 6:**
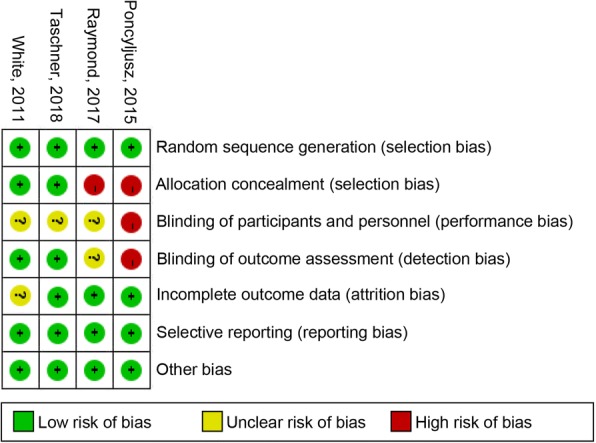
Risk of bias: A summary table for each risk of bias item for each study

The details of the risk bias for all studies were shown in Fig. 6. There were two clinical trials that have a higher risk of bias in allocation concealment. For the blinding of participants and personnel, the risk of bias was high in one study and the other three trials were unclear. For the blinding of outcome assessment, the risk of bias was high in one study and unclear risk of bias in another trial. For incomplete outcome data, the risk of bias was unclear in one trial. Apart from these four items, there were no high or unclear risk of bias in any of the other items was observed.

## Discussion

Hydrogel coils embolization for intracranial aneurysm might be superior to bare platinum coils in mid-term outcome based on the evidence from our current meta-analysis. We discovered that hydrogel coils had no distinct benefit in periprocedural efficacy and safety outcomes, including initial post-operational complete occlusion, residual aneurysm neck, residual aneurysm, failed procedure, coil migration, perforation, thromboembolic complication, hydrocephalus, morbidity and mortality, which was in accordance with other studies [4, 9, 16, 17, 19, 21, 23, 24].

Hydrogel coils had a tendency of decreasing mid-term residual aneurysm neck (*P* = 0.054) and exhibited valid preventions from the mid-term residual aneurysm (*P* = 0.002). Meanwhile, as a potential tendency of a favorable outcome, no significant differences in the numbers of mid-term complete occlusion were detected (RR 1.13, 95% CI 0.98 to 1.31, *P* = 0.094). Although the sensitivity analysis demonstrated that the consolidated statistics about mid-term complete occlusion were stabilized, the results might be influenced by the high heterogeneity. Subgroup analyses depicted that second-generation hydrogel coils could improve complete occlusion (*P* = 0.005) compare to first-generation coils (*P* = 0.450) when hydrogel coil was detached.

In addition, recurrence was a high-profile and controversial issue among different clinical trials and systematic reviews: HELPS (Hydrocoil Endovascular aneurysm occLusion and Packing Study) [25–27], GREAT(German-French Randomized Endovascular Aneurysm Trial) [15, 20] and Serafin’s systematic review [4] illustrated that hydrogel coils resulted in a lower rate of recurrence. In contrast, no significant differences by using hydrogel coils in terms of aneurysm recurrence were observed through Poncyljusz’s RCT [17] and PRET (Patients prone to Recurrence after Endovascular Treatment) [21, 28, 29]. In our meta-analysis, we pooled 1526 patients from 4 RCTs and drew a conclusion that hydrogel coils showed impacts upon avoiding recurrence (RR 0.78, 95% CI 0.65 to 0.94, *P* = 0.008) against bare platinum coils. Meanwhile, we also found that the number of patients without intracranial aneurysm recurrence in DSA had no distinct difference for the two groups (RR 1.06, 95% CI 0.99 to 1.13, *P* = 0.106). A hypothesis was established to explain the question that some recurrent patients was not diagnosed with aneurysm recurrence by angiography but through other ways such as CTA, MRA or clinical manifestations [30].

Surprisingly, bare platinum coils group contrarily had an advantage over hydrogel coils group in mid-term patients’ good functional outcome, which is defined as the mRS score from 0 to 2. Why did bare platinum coils group have a favorable result of mid-term patients’ good functional outcome? Although the statistics revealed that hydrogel coils could prevent intracranial aneurysm patients from mid-term residual aneurysm, recurrence; we suspect that it was the degree of damage at the aneurysm ruptured point and post-operational complications that have more influences on the patients’ prognosis which could be measured by mRS score [31]. Hence, we performed a subgroup analyses between high ruptured rate subgroup (*N* > 30%) and low ruptured rate subgroup (*N* < 30%). We found no significant differences in good functional outcome when we separated high ruptured rate trials from low ruptured rate trials. Therefore, the favorable outcome of bare platinum coils could be the result of the selection bias of patients in trials and degree of baseline damage patients between hydrogel coils group and bare platinum coils group.

We were also interested in the reasons of why there were no significant differences in initial retreatment (*P* = 0.675) between the two groups in spite of the fact that bare platinum coils had a distinctly higher rate of recurrence than hydrogel coils (*P* = 0.008). We found that compared with recurrence, the number of retreatment was obviously smaller and it meant only a part of recurrent patients chose or had a chance to retreat. Therefore, we assumed that the small amount of retreatment was not able to get a statistically significant difference under the circumstance that the number of total patients remained relatively large.

Subgroup analyses indicated that second-generation hydrogel coils could improve complete occlusion and reduce residual aneurysm neck from the results of the mid-term follow-up. We believe it is because that second-generation hydrogel coils were only abundantly employed in GREAT 2018, which could cause the favorable results of hydrogel coils. In the further, when second-generation hydrogel coils are used in more clinical trials, we could get a more reliable outcome of it. The other subgroup analysis found that the high ruptured rate subgroup had a higher probability to get occluded completely. The mechanisms of coils embolization therapy were to form a thrombus in the aneurysm by coils attracting negatively charged blood components (red blood cells, white blood cells and platelets, etc.) to coagulate [32]. Hence, hyperfunction of coagulation system under stress might result in the favorable complete occlusion in high ruptured rate subgroup.

On the basis of our knowledge, it was the first meta-analysis about the comparison between hydrogel coils and bare platinum coils, using evidence solely from RCTs (randomized clinical trials). Previous systematic reviews and meta-analysis were predominantly or entirely based on non-randomized researches [4, 9]. Combining the findings from uncontrolled trials results in a heterogeneous dataset, therefore, these systematic reviews are flawed. Additionally, some systematic reviews and meta-analysis were comprised of not only hydrogel coils but also other bioactive coils [23, 25]. Which resulted in an analysis including mixed types of coil; the outcome of these reviews and meta-analysis inevitable have deviations. In spite of some subgroup analyses about hydrogel coils performed in these research studies [23], the comprehensiveness of the comparison between hydrogel coils and bare platinum coils was insufficient on account of the included trials’ quantity [23]. Different from above-mentioned systematic reviews, all patients in our present meta-analysis were intervened by either hydrogel coils or bare platinum coils in randomized trials, which was the best method to divide risk factors equally over the two groups [33]. Limitations of our meta-analysis should be noticed. First, this meta-analysis was performed on the foundation of limited statistics. We only pooled 4 published RCTs [16, 17, 19, 21] with 1526 patients (hydrogel, *n* = 767; bare, *n* = 759) to examine the efficacy and safety of hydrogel coils vs. bare platinum coils for intracranial aneurysm. Additionally, the included RCTs showed heterogeneity in the data of periprocedural mortality (*I*^2^ = 63.8%), mid-term mortality (*I*^2^ = 62%) and mid-term complete occlusion (*I*^2^ = 49.6%). The sensitivity analysis demonstrated that all the consolidated statistics were stabilized, but these disadvantages of the included studies could not be neglected. Lastly, in spite of the patients being randomized in 4 RCTs, the heterogeneous risk factors were still noticeable, and the baseline damage degree or mRS scores between hydrogel coils group and bare platinum coils group might vary patient by patient.

## Conclusions

In conclusion, our meta-analysis demonstrated that endovascular treatment for intracranial aneurysms by hydrogel coils had preventive efficacy on mid-term recurrence and residual aneurysm, but didn’t show any significant differences in other outcomes. Second-generation hydrogel coils might exhibit potential favorable impacts on mid-term complete occlusion and residual aneurysm neck, and therefore could affect clinical outcome. Based on our findings, we suggest future researchers to consider testing the possible therapeutic effect of second-generation hydrogel coils in patients with intracranial aneurysms.

## Additional file

Additional file 1: The sensitivity analysis showed that all of the consolidated results were stable. **Figure S1.** Fig. 3 C Sensitivity analysis of Periprocedural mortality from 4 RCTs. **Figure S2.** Fig. 4 A Sensitivity analysis of Mid-term complete occlusion from 4 RCTs. **Figure S3.** Fig. 5 G Sensitivity analysis of Mid-term mortality from 4 RCTs. (DOCX 281 kb)

## Abbreviations

CI: Confidence interval
DSA: Digital subtraction angiography
GDC: Guglielmi detachable platinum coils
GREAT: German-French Randomized Endovascular Aneurysm Trial
HELPS: Hydrocoil Endovascular aneurysm occLusion, and Packing Study
HES: Hydrogel embolic system
ISAT: International Subarachnoid Aneurysm Trial
mRS: modified Rankin Scale
PRET: Patients prone to Recurrence after Endovascular Treatment
RCT: Randomized controlled trials
RR: relative risk
