# Supplementary material for: Hydrogel coils versus bare platinum coils for the endovascular treatment of intracranial aneurysms: a meta-analysis of randomized controlled trials
Source: BMC Neurol. 2018 Oct 5;18:167. doi: 10.1186/s12883-018-1171-8 (PMC6172718; doi:10.1186/s12883-018-1171-8)
Supplement: Supplementary file 1 — The sensitivity analysis showed that all of the consolidated results were stable. Figure S1. Fig. 3 C Sensitivity analysis of Periprocedural mortality from 4 RCTs. Figure S2. Fig. 4 A Sensitivity analysis of Mid-term complete occlusion from 4 RCTs. Figure S3. Fig. 5 G Sensitivity analysis of Mid-term mortality from 4 RCTs. (DOCX 281 kb) [file 12883_2018_1171_MOESM1_ESM.docx]

**Title: Hydrogel Coils versus Bare Platinum Coils for the Endovascular Treatment of Intracranial Aneurysms: a Meta-analysis of Randomized Controlled Trials****.**

Tao Xue^1,#^, Zhouqing Chen^1^^,#^, Weiwei Lin^2^, Jiayi Xu^3^, Xuming Shen^4,^*, Zhong Wang^1,^*

^1^ *Department of Neurosurgery & Brain and Nerve Research Laboratory,* *The First Affiliated Hospital of Soochow University,* *Suzhou, Jiangsu Province, 215006, China*

^2^ *University of Pittsburgh School of Pharmacy, Pittsburgh, PA, 15219, USA*

^3^ *Department of Ophthalmology, The First Affiliated Hospital of Soochow University, Suzhou, Jiangsu Province, 215006, China*

^4^ *Department of Neurosurgery,* *Taicang Affiliated Hospital of Soochow University, Suzhou, Jiangsu Province, 215400, China*

**The sensitivity analysis showed that all of the consolidated results were stable**

**Figure S1**

**Fig. 3 C Sensitivity analysis of Periprocedural mortality from 4 RCTs:
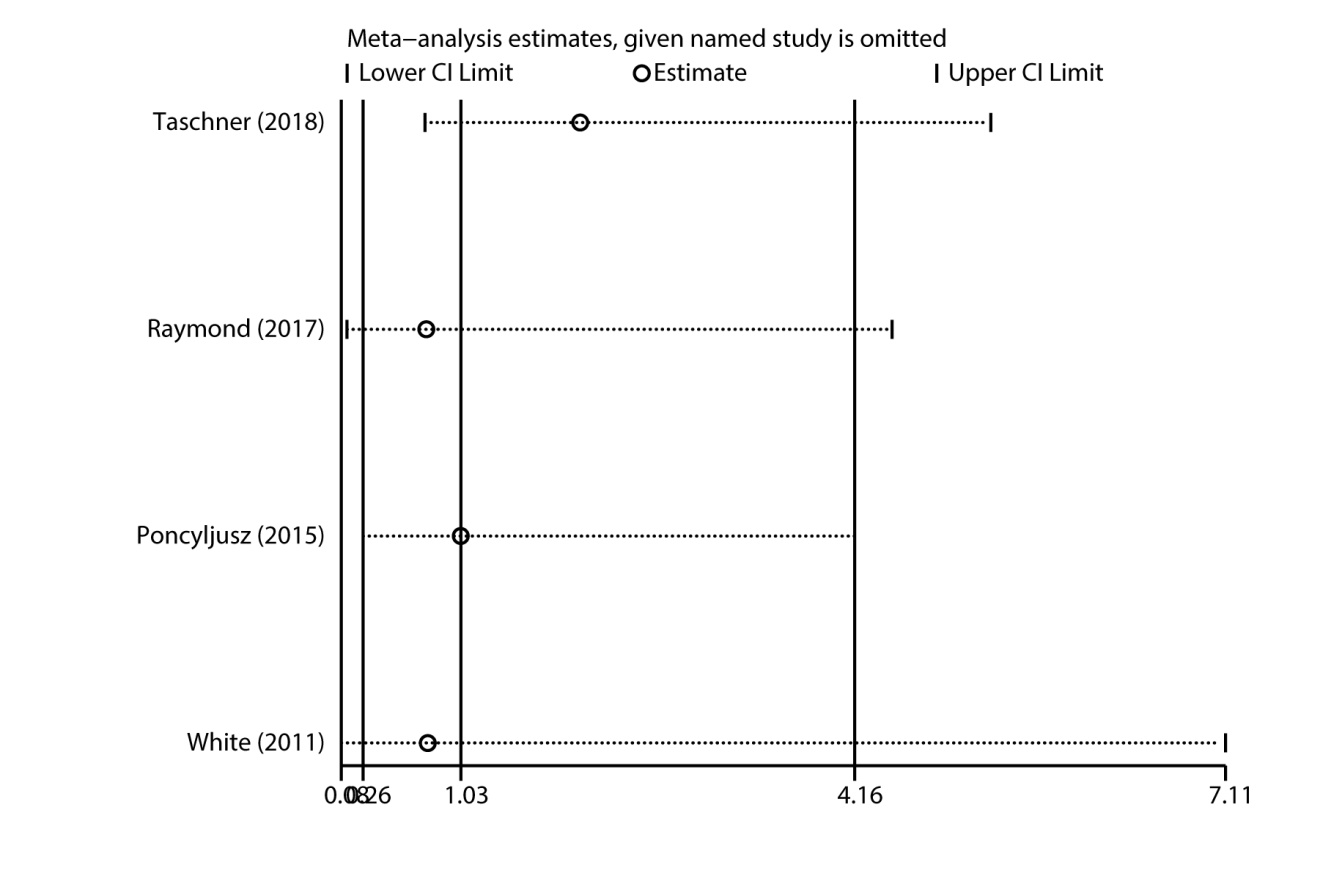
**

**Figure S2**

**Fig. 4 A Sensitivity analysis of** **Mid-term complete occlusion from 4 RCTs:**

**
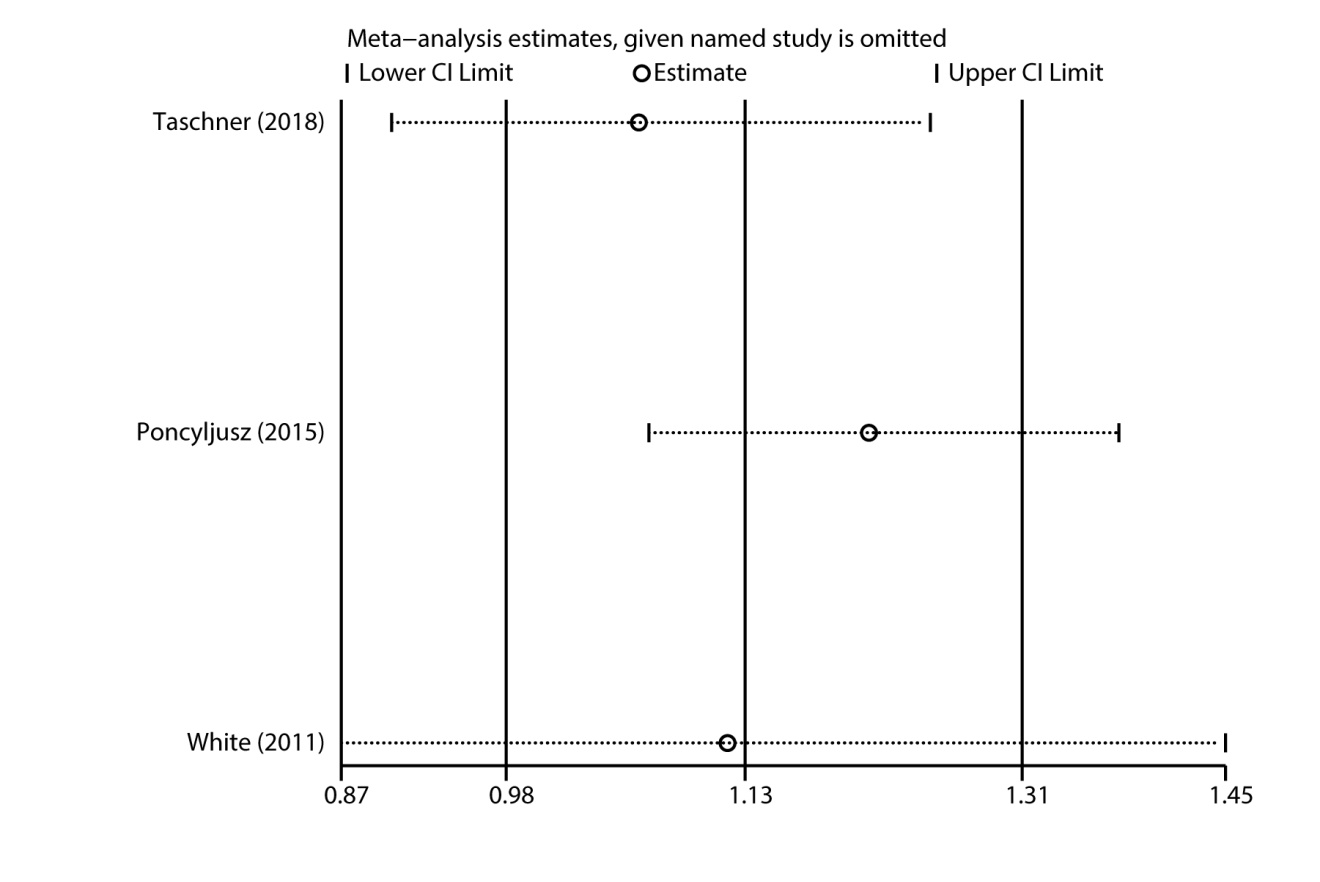
**

**Figure S3**

**Fig. 5 G Sensitivity analysis of Mid-term mortality from 4 RCTs:**

**
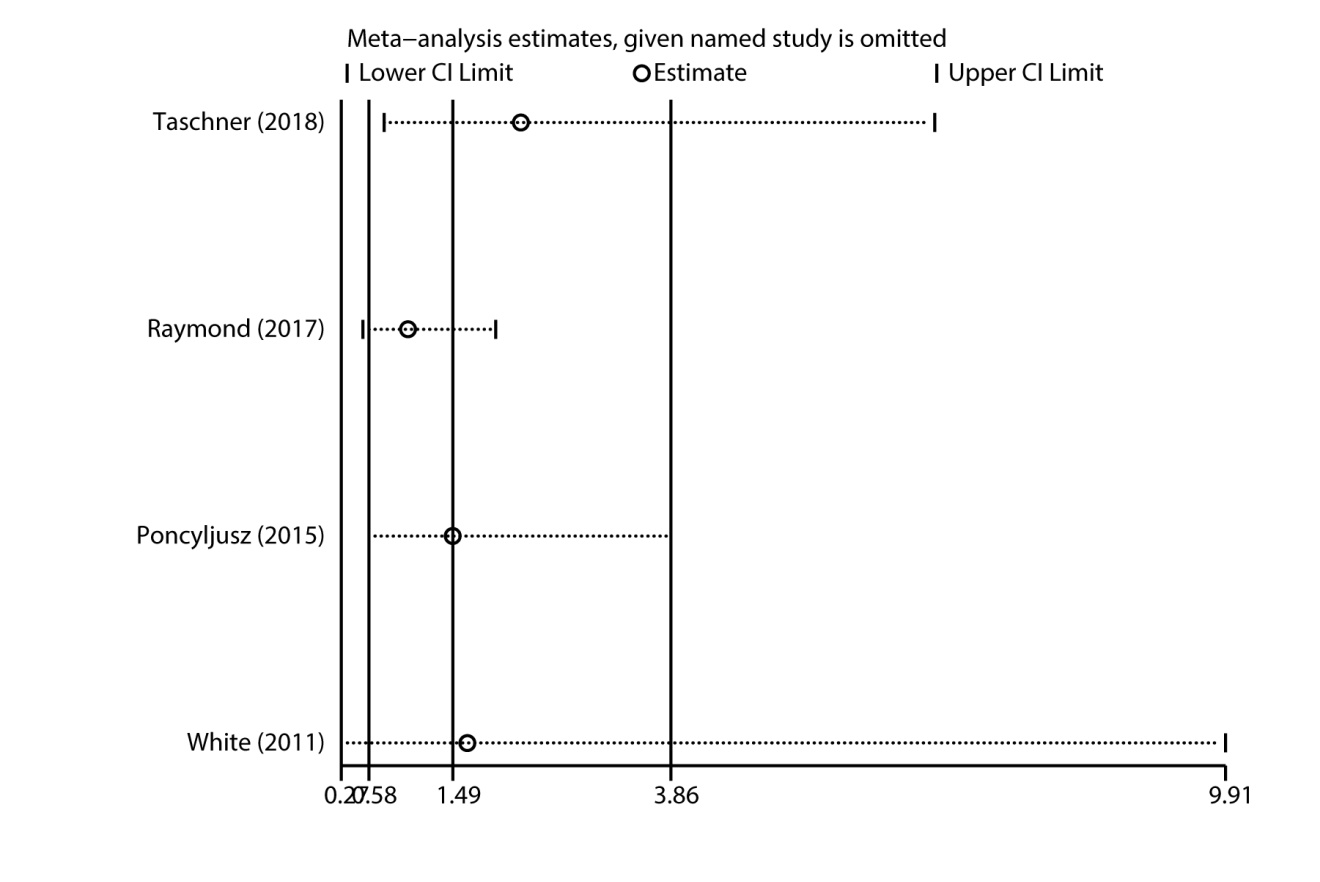
**
